# Effects of testosterone and flutamide on reproduction in *Brachionus calyciflorus*

**DOI:** 10.1038/s41598-017-05517-4

**Published:** 2017-07-26

**Authors:** Jian Tian, Lulu Liu, Yajie Han, Yuanhao Yang, Sichen Jin, Jiaxin Yang

**Affiliations:** School of Life Sciences, Nanjing Normal University, Nanjing, Jiangsu 210023 China

## Abstract

The effects of testosterone and flutamide on reproduction in *Brachionus calyciflorus* were studied. Asexual reproduction in *B. calyciflorus* was not affected by testosterone at different concentrations of flutamide. Flutamide in combination with 0, 25, 50, or 75 µg L^−1^ testosterone had a significant effect on mixis rate. The combination of 5 µg L^−1^ flutamide with 25 µg L^−1^ or 50 µg L^−1^ testosterone resulted in a mixis rate that was 2.2× lower than that with flutamide alone. Fertilization rate was significantly decreased by 7.5 µg L^−1^ flutamide in combination with 25, 50, or 75 µg L^−1^ testosterone. The number of resting eggs produced per mictic female was significantly lower at all concentrations of testosterone. A low concentration of flutamide in combination with testosterone resulted in antagonism, increasing the number of resting eggs produced. However, when testosterone was combined with a higher concentration of flutamide, resting egg production declined. Therefore, long-term exposure to either testosterone, flutamide, or a combination of these two compounds may significantly reduce resting egg production in rotifers. This implies that resting egg production is affected differently by hormone pathways.

## Introduction

Anthropogenic chemicals with the potential to perturb the function of endocrine systems are called endocrine disrupting chemicals (EDCs). Many EDCs can interfere with reproduction, development, survival, distribution, and cause disease or deformity in organisms and their offspring^1^. Previous studies have demonstrated that some anthropogenic chemicals can disrupt normal hormonal communication, producing harmful effects on reproduction in a wide variety of animals, including rotifers^2–4^. In respect to endocrine disruptors, rotifers seem to be particularly sensitive to androgenic and anti-androgenic substances^5^. And especially *Brachionus calyciflorus*, are ideal models for ecotoxicology studies because of their wide distribution, short life history, facility of culture, and the convenience of resting eggs^6^. *Monogonont* rotifer reproduction includes asexual and sexual periods. In asexual reproduction, an amictic female rotifer produces clones of herself via ameiotic parthenogenesis, whereas in sexual reproduction, females produce haploid eggs that develop into males if unfertilized or into resting eggs if fertilized. Dormant resting eggs ensure the survival of the species during adverse environmental conditions^7^. The initiation of sexual reproduction, therefore, is a critical phase of the rotifer life cycle that has important ecological and evolutionary consequences for the population. The adaptive features of resting eggs are outlined including their contribution to genetic variability through recombination, their provision for environmental escape by dormancy^8^. The rotifer life cycle, including both isolated parts and the full life-cycle, has been used for the development of toxicity tests to assess the hazards of anthropogenic chemicals in freshwater and marine environments^9^. Examples of the application of rotifers in toxicity testing include measurements of 24 h mortality for comparing the toxicity of chemicals^10^, the Triclosan exposure on the hatching rate during the formation period had a greater effects on those during the resting egg hatching period^11^, 2 d population growth rate (r) for assessing chronic toxicity^12^, life history parameters for the assessment of the toxicity^13^, full life cycle toxicity assessment using rotifer resting egg production, a 96-h *B. calyciorus* resting egg toxicity test was developed and used to estimate the toxicity of pentachlorophenol (PCP) and copper. Results were compared to a variety of acute and sublethal endpoints for both toxicants^9^, and mixis rate and hatchability of resting eggs for the detection of the effects of pesticide exposure^14^. Besides, rotifers may be more sensitive than the standard invertebrate test species *Daphnia magna* to fungicides^15^.

The effects of various hormones on rotifer reproduction have been recently investigated^16^. Batch cultures of *Brachionus plicatilis* treated with 0.05 and 0.5 mg L^−1^ juvenile hormone (JH) experienced a significant increase in mixis rate of approximately 30%, compared to a 10% mixis rate in the control. However, 50 mg L^−1^ JH decreased the mixis rate compared to the controls. Treatment of rotifers with 50 mg L^−1^ 17β-estradiol increased the mixis rate to approximately twice that of the control, although lower concentrations had no effect. Growth hormone and gamma aminobutyric acid significantly stimulated population growth and mictic female production over control levels in *B. plicatilis* ^17^. Treatment of female rotifers with JH (5 and 50 mg L^−1^) resulted in a significantly higher mictic rate that increased from 4% in the controls to 8% in the F_2_ generation of females in *B. plicatilis* ^18^. Progesterone at 10 mg L^−1^ significantly increased the mixis rate (1.18 times higher than the control) of *B. manjavacas* ^19^. A depression of the fertilization rate in *B. calycifloru*s was reported following treatment with 1 µg mL^−1^ nonylphenol, 10 µg mL^−1^ testosterone, or 50 µg mL^−1^ flutamide^4^. In addition, population growth rate and the ratio of ovigerous to non-ovigerous females are both suitable endpoints for the detection of the reproductive disrupting effects of ethinylestradiol, nonylphenol, and testosterone^20^. Thus, rotifer is one of the promising tools for the assessment of the impact of potential endocrine disruptors on aquatic invertebrates^21^. However, many of these previous studies have only investigated the limited effects of single hormones in isolation on rotifer reproduction. The combined effects of these hormones on rotifer reproduction have not yet been studied.

In this study, testosterone (an androgen) and flutamide (an anti-androgen) were selected to test the hypothesis that these chemicals would have antagonistic effects on rotifer reproduction. To test this hypothesis, the effects of testosterone in the presence of different concentrations of flutamide on *B. calyciflorus* reproduction was investigated.

## Materials and Methods

### Test animals

*Brachionus calyciflorus* were obtained by hatching resting eggs which were donated by Prof. T.W. Snell (Georgia Institute of Technology). This rotifer strain was produced in a lab from a population originally collected in Gainesville, Florida, USA, in 1983. Hatching was performed in synthetic freshwater (EPA medium; 96 mg NaHCO_3_, 60 mg CaSO_4_·H_2_0, 123 mg MgSO_4_, and 4 mg KCl in 1 L of deionized water, Table 1) adjusted to pH 7.5 at 25 °C under a light intensity of 2000 lux^12, 22^. After 18 h of hatching, cysts were inspected to ensure the collection of test animals within 2 h of hatching. Rotifers were fed with green *Chlorella pyrenoidosa* (Institute of Hydrobiology, Chinese Academy of Sciences, Wuhan, China) of 3.3 × 10^6^ cell per ml that was cultured in continuous culture using HB-4 medium^23^. Algae were grown at a high density and were centrifuged and diluted to a suitable density in EPA medium prior to feeding.

**Table 1:** Concentration modifications from ASTM.

	Reagent added (mg/L)			
	NaHCO_3_	CaSO_4_·2H_2_O	KCL	MgSO_4_
ASTM	96.0	60.0	4.0	60.0
Modifications	96.0	60.0	4.0	123.0

### Test chemicals

Stock testosterone and flutamide solutions of 1000 mg L^−1^ (analytical standard 99.5% and 99.8%, respectively, Sigma–Aldrich chemical Chemie, Munich, Germany) were prepared by dissolution in 100% acetone (Analytical grade, 99.5%). For experimental exposures, testosterone and flutamide stock solutions were further diluted in EPA medium with a final acetone concentration less than 0.01%. This concentration of acetone was demonstrated to not have any significant influence on the rotifer survival, growth and reproduction parameters compared to the controls^19^.

### Experiment design

A number of range-finding experiments were performed to determine the lowest observed effect concentrations of testosterone (100 μg L^−1^) and flutamide (10 μg L^−1^) on the population growth rate at 2 d^12^. Based on these results, exposure concentrations were selected for flutamide (F; 0, 2.5, 5.0, and 7.5 µg L^−1^) and testosterone (T; 0, 25, 50, and 75 µg L^−1^), and these were combined to obtain 16 treatment combinations: F_0_(0 + 0, 0 + 25, 0 + 50, 0 + 75 µg L^−1^), F_1_(2.5 + 0, 2.5 + 25, 2.5 + 50, 2.5 + 75 µg L^−1^), F_2_(5.0 + 0, 5.0 + 25, 5.0 + 50, 5.0 + 75 µg L^−1^), F_3_(7.5 + 0, 7.5 + 25, 7.5 + 50, 7.5 + 75 µg L^−1^).

### Asexual population growth rate

Four young rotifers (less than 4 hours old) were introduced into 8 mL of EPA medium containing 3.0 × 10^6^ cells mL^−1^ *C. pyrenoidosa* (freshwater) in a 16 × 150 mm glass test tube. There were five replicate tubes at each treatment combination for a total of 80 test tubes. The test tubes were placed on a rotator turning at 10–15 rph at 25 °C. After 48 h, the total number of rotifers in each tube was counted. After each count, the volume of each tube was restored to its original level. The population growth rate (r) was calculated according to the exponential growth equation: r = (ln N_t_ − ln N_0_)/t, where N_t_ = the final number of female rotifers in the tube, N_0_ = the initial number of rotifers in each tube, and t = time in days^24^. After counting, the rotifers were placed back into the tubes and cultured for two more days^12^.

After 4 d, the number of each type of female rotifer in the tubes was counted following the methods described by Preston^4^. From these counts, the mixis rate (MR) was calculated for each test tube as the proportion of mictic females among the ovigerous females, and the fertilization rate (FR) was calculated as the proportion of fertilized mictic females among the mictic females. The ratio of ovigerous fertilized females was calculated as fertilized females among all rotifers. After counting, rotifers and resting eggs were placed back into the original tubes and continuously cultured until 7 d^4^. At 7 d, the number of resting eggs in each test tube was counted to compare the effect of the treatments on resting egg production^14^.

### Statistical analysis

All statistical analyses were performed using SPSS 16.0^25^. The effects of testosterone and flutamide were compared using two-way analys is of variance (ANOVA), with concentrations as the independent variables, and population growth rate (r), mixis rate (MR), fertilization rate (FR), or RE as the dependent variable. Tukey’s test was used for pairwise comparisons of each treatment concentration relative to the control.

## Results

### Population growth rate

**Figure 1:**
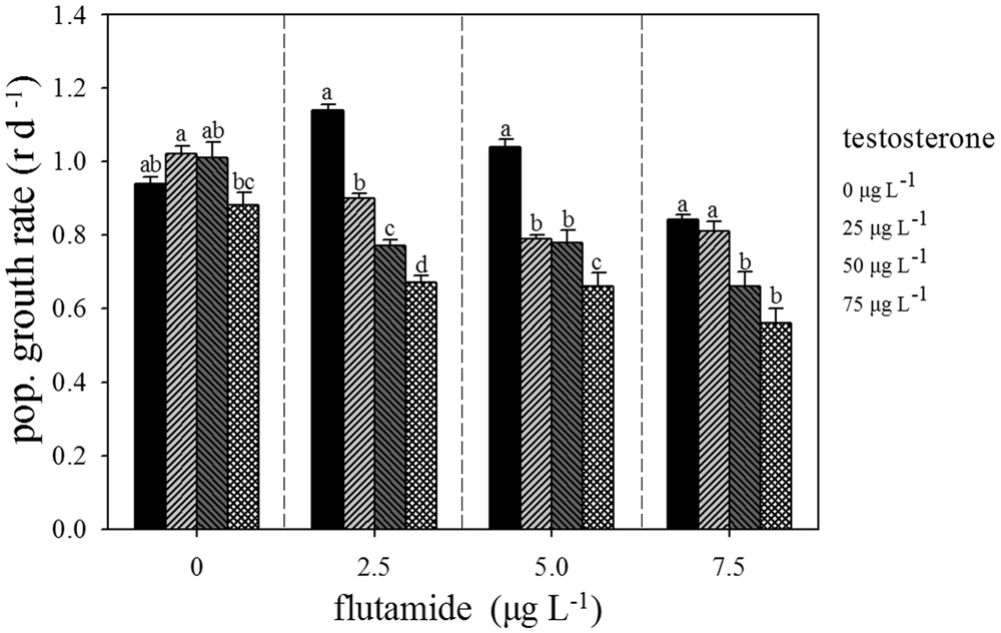
Effects of testosterone and flutamide on the population growth rate (r) in *Brachionus calyciflorus* (n = 5).

The addition of testosterone alone significantly affected the population growth rate (r) (F_3,1_ = 3.55, p = 0.039) (Fig. 1). Testosterone at 25 µg L^−1^ resulted in a significant increase in the population growth rate relative to the control (p < 0.05), while at 50 µg L^−1^ this effect was not significant (p > 0.05). With the addition of 2.5 µg L^−1^ flutamide, the population growth rate was significantly decreased with increases in the concentration of testosterone (F_3,16_ = 107.55, p = 0.000). Flutamide concentrations of 5.0 and 7.5 µg L^−1^ added to the different concentrations of testosterone significantly affected the population growth rate (F_3,16_ = 27.36, p = 0.000, F_3,16_ = 114.88, p = 0.000, respectively). The population growth rate was increased at a low concentration of flutamide in combination with testosterone, while it was reduced at higher concentrations of flutamide. This pattern suggest a hormetic effect^26^. Flutamide mixed with testosterone significantly decreased the population growth rate.

Treatment with testosterone or flutamide alone significantly affected the population growth rate (F = 31.35, p = 0.000, and F = 8.555, p < 0.05, respectively), but the combination of these treatments did not have any further significant effect on the population growth rate (F = 1.87, p > 0.05, Table 2).

**Table 2:** Summary of two-way ANOVA on the interaction between flutamide concentration (0, 2.5, 5.0, 7.0 μg L^−1^) and testosterone concentration (0, 25, 50, 75 μg L^−1^) on population growth rate, mixis rate, fertilization rate, number of resting eggs produced of *B. calyciflorus* in each tissue (n = 5).

Parameters	Source of variation	DF	F	P
Pop. growth rate	flutamide	2	8.555	<0.001
	Testosterone	2	31.348	<0.001
	flutamide × testosterone	4	1.876	0.138
Mixis rate	flutamide	2	35.163	<0.001
	Testosterone	2	89.410	<0.001
	flutamide × testosterone	4	14.644	<0.001
Fertilization rate	flutamide	2	15.038	<0.001
	Testosterone	4	105.575	<0.001
	flutamide × testosterone	4	1.368	0.264
Resting eggs produced	flutamide	2	27.301	<0.001
	Testosterone	2	210.758	<0.001
	flutamide × testosterone	4	10.644	0.180

### Rotifer mixis rate at 4 d

**Figure 2:**
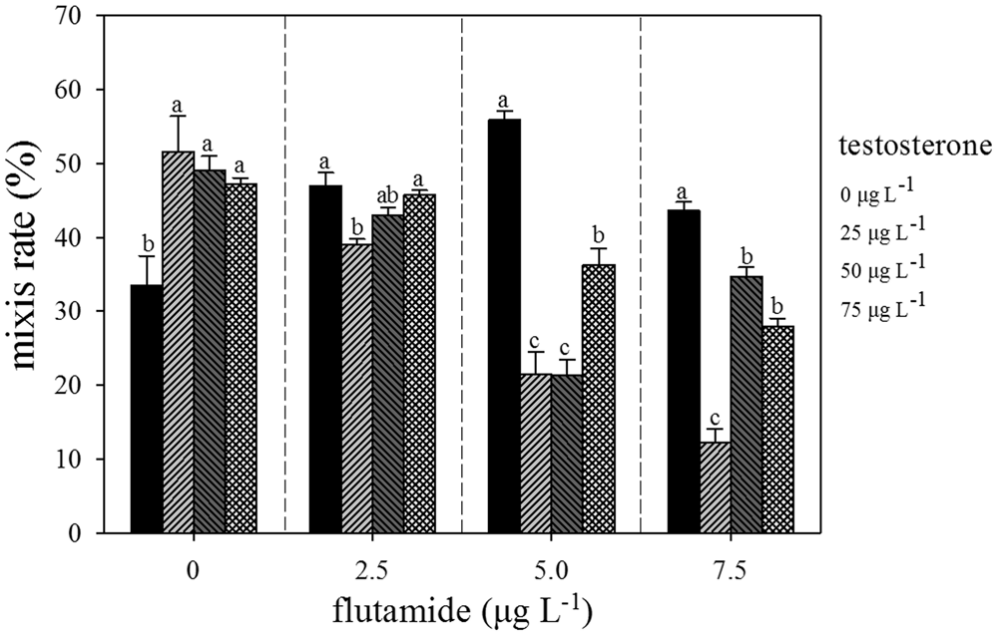
Effects of testosterone and flutamide on the mixis rate (MR) at 4 d in *B. calyciflorus* (n = 5).

The addition of testosterone significantly affected the mixis rate (MR) (F_3,16_ = 5.583, p = 0.008) (Fig. 2). Testosterone treatment significantly increased the MR relative to the control (p < 0.05), however, there were no significant differences between the different concentrations of testosterone. The addition of 2.5 µg L^−1^ flutamide to the 0, 25, 50, and 75 µg L^−1^ testosterone groups significantly affected the MR (F_3,16_ = 7.477, p = 0.002). The MR at 25 µg L^−1^ testosterone was the lowest, while at 50 and 75 µg L^−1^ testosterone the MR was similar to the control. Other groups showed similar results. However, 5 µg L^−1^ flutamide, in combination with 25 or 50 µg L^−1^ testosterone, resulted in the largest reduction in the MR. When rotifers were exposed to testosterone or flutamide alone, the MR was significantly affected (F = 89.41, p < 0.05, and F = 35.16, p < 0.05, respectively). However, there was also a significant interaction between the effects of testosterone and flutamide on the MR (F = 14.64, p < 0.05, Table 2).

### Fertilization rate at 4 d

**Figure 3:**
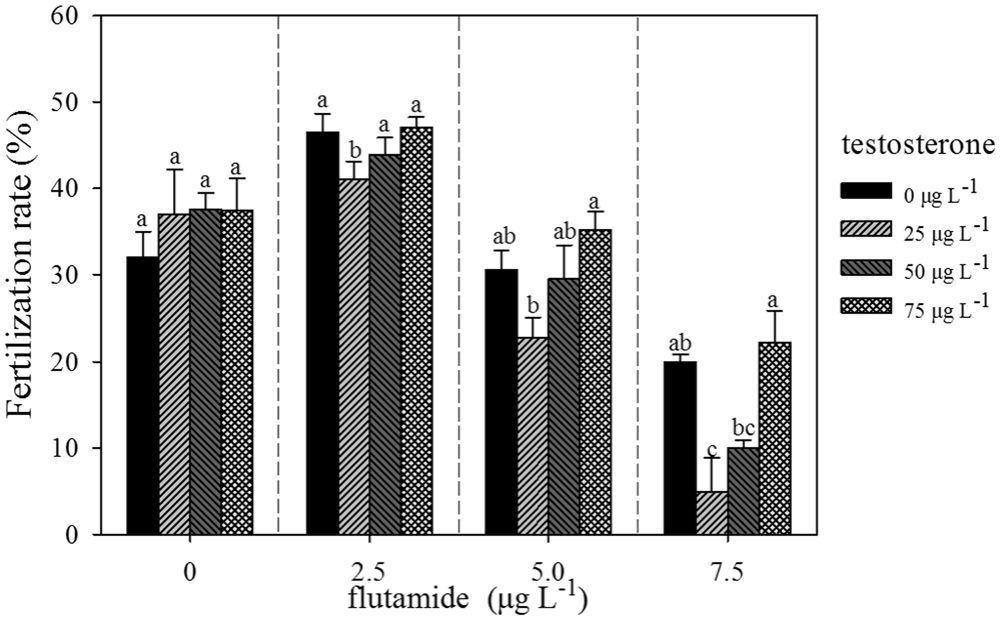
Effects of testosterone and flutamide on the fertilization rate in *B. calyciflorus* (n = 5).

Flutamide at 2.5 or 5.0 µg L^−1^ in combination with different concentrations of testosterone did not have any significant effect on the Fertilization Rate (FR), but a significant effect was observed at 7.5 µg L^−1^ flutamide (F_3,16_ = 0.469, p = 0.708, F_3,16_ = 1.180, p = 0.186, F_3,16_ = 2.981, p = 0.063, and F_3,16_ = 10.55, p = 0.000 for 0, 2.5, 5.0, and 7.5 µg L^−1^, respectively) (Fig. 3). In addition, treatment with 2.5 µg L^−1^ flutamide tended to increase the Fertilization Rate (FR) relative to the control, however, higher concentrations of flutamide (5 and 7.5 µg L^−1^) resulted in a declining trend in FR. With two-way analysis of variance, testosterone or flutamide alone significantly affected the FR (F = 105.87, p < 0.005, and F = 15.04, p < 0.005, Table 2), however, the combined effect of these treatments had no further significant effect on the FR (F = 1.37, p > 0.05, Table 2).

### Number of resting eggs produced at 7 d

**Figure 4:**
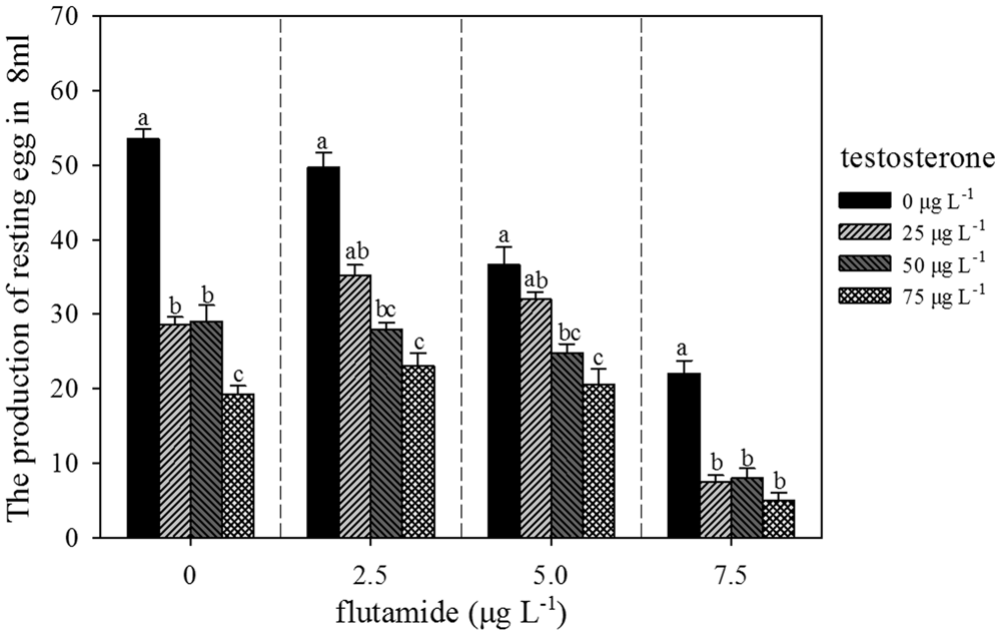
Effects of testosterone and flutamide on the number of resting eggs produced at 7 d in *B. calyciflorus* (n = 5).

The addition of testosterone significantly decreased the number of resting eggs produced in the absence of flutamide (F_3,16_ = 78.47, p = 0.000) (Fig. 4). At 2.5, 5.0, and 7.5 µg L^−1^ flutamide, the results were similar to the controls across the different testosterone concentrations. However, at 7.5 µg L^−1^ flutamide and 25 µg L^−1^ testosterone, the number of resting eggs produced was significantly lower than the control (p < 0.05). With two-way analysis of variance, testosterone alone, flutamide alone, and the combination these treatments significantly affected the number of resting eggs (F = 21.75, p = 0.000, F = 27.31, p = 0.000, and F = 3.43, p < 0.05, Table 2).

## Discussion

In recent years, a new field of scientific inquiry has emerged against the backdrop of numerous reports of disrupted endocrine function in various groups of vertebrates. U.S Environmental Protection Agency (EPA) lists sixty kinds of the environmental hormone substances. These included testosterone and flutamide. It has not been reported in China in recent years. Rotifers were chosen to assess the impact of potential endocrine disruptors on invertebrate reproduction in this study. It was observed that low concentration of testosterone increased the rotifer population growth rate but a higher concentration reduced the population rate, which was similar to a previous study^20^. It is probably because a slight stress can increase the reproduction of many animals due to compensatory response as hormesis, while a severe stress might suppress it ref. 27. In another study, medroxyprogesterone had a different effect than progesterone on the population growth of *B. manjavacas* ^19^. Snell and Moffat (1992) reported that a 2 d population growth test with *B. calyciflorus* was often more sensitive than a 7 d *Ceriodaphnia dubia* reproductive test. In the present study, the results of the combined effect of flutamide and testosterone on *B. calyciflorus* also demonstrated that the 2 d population growth rate might be a sensitive parameter. A significant reduction in the number of resting eggs was observed after 7 d with increasing concentrations of testosterone in the current study. These results were similar to those reported by Marcial *et al*.^14^ who observed a decrease in resting egg production in *B. plicatilis* exposed to pesticides including diazinon, fenitrothion, methropone, and isoprothiolane. These results infer that testosterone exposure in the current study may have disrupted reproduction in *B. calyciflorus* resulting in a reduction in the formation of resting eggs, similar to that reported by Marcial *et al*.^14^. The reduced egg production following testosterone treatment in this study was antagonized by a low concentration of flutamide, which resulted in an increased resting egg production. However, when testosterone was combined with a higher concentration of flutamide, the resting egg production declined. This result was similar to that observed in *B. manjavacas* exposed to medroxyprogesterone and juvenile hormone, but different to that observed following progesterone exposure^19^.

From the results of the two-way ANOVA, it was apparent that testosterone and flutamide combined had antagonistic effects on the population growth rate of *B. calyciflorus*. A similar result was observed for the mixis rate following the combined exposure. However, testosterone and flutamide combined did not have antagonistic effects on the fertilization rate. These results suggest that the fertilization rate is not a sensitive parameter for the assessment of the effects of testosterone and flutamide on rotifers. Exposure to progesterone, combined with either testosterone or estradiol at 1000 μg L^−1^, resulted in a decrease in resting egg production *B. calyciflorus* ^16^. In the current study, the 2 d population growth rate and the fertilization rate at 4 d were more sensitive than the number of resting eggs produced after 7 d. In addition, Rotifers is a good model for studies of the effects of endocrine disruptors on reproduction. The observation that testosterone combined with flutamide resulted in a decreased resting egg production may be explained by the observation that *B. calyciflorus* may use oxidized sterols to regulate sexual reproduction^16^.

Rotifers may provide a better model than other animals for studies of the effects of endocrine disruptors on reproduction. The molecular mechanisms of steroid action have been investigated in rotifers. The presence of a steroid signaling cascade in *B. manjavacas* that regulates sexual reproduction was suggested, and exposure to 10 mg L^−1^ progesterone resulted in a 4.4× increase in resting egg production in this species^19^. The effects of anti-androgenic compounds on sexual reproduction in *B. calyciflorus* were investigated by Joaquim-Justo and Snell. In the current study, 25 µg L^−1^ testosterone resulted in a resting egg production that was lower than the control (1.8×), and exposure to higher concentrations of testosterone had a similar result. Exposure to high concentrations of flutamide and testosterone have been demonstrated to increase population growth of *B. plicatilis* ^4, 17^ demonstrated that flutamide and testosterone may interfere with endocrine signaling, causing a reduction in resting egg production in rotifers. In the current study, 7.5 µg L^−1^ flutamide combined with testosterone had a stronger effect on 7 d resting egg production than flutamide or testosterone alone, indicating that rotifer resting egg production may be the most sensitive endpoint^28^. Testosterone and flutamide may have their effects on reproduction in the rotifer in the same way, possibly through the same rotifer steroid receptor. In a previous study, the percentage of fertilized ovigerous female rotifers was reduced to 11% versus 26% in the controls following treatment with 10 µg L^−1^ testosterone, while 1 µg L^−1^ flutamide significantly reduced fertilization to 1% versus 14% in controls^4^. However, in the current study, testosterone up to 75 µg L^−1^ had no effect on the FR, and low concentrations of flutamide increased the FR while high concentrations reduced the FR. The reasons for the differences between these studies may be due to different culture and experimental methods.

The progesterone receptor has been described in female and male *B. manjavacas* and has been demonstrated to bind progesterone^29^. Existing evidence suggests that steroid hormones have a role in endocrine signaling in invertebrates^26^. Vertebrate-type steroids have been detected in several arthropod species^30^, but the functional role of these hormones is still not clear. A rotifer progesterone receptor has been discovered^19^, but an estrogen receptor has not been found in the rotifer transcriptome and there is only weak evidence for the presence of an androgen receptor. Transcriptome surveys have shown that steroid receptors, biosynthetic enzymes, and signal transduction pathway elements are present in brachionids. There is strong evidence that steroids similar to progesterone and androgens are employed as regulatory signals in rotifers, whereas estrogens seem less important^31^.

The effects of steroid hormones on reproduction in other invertebrates have been reported. Long-term testosterone exposure at concentrations ranging from 0.31 to 2.48 mg L^−1^ in *Daphnia magna* resulted in reduced fecundity and fertility, and the decrease in fecundity was associated with an increase in the number of aborted eggs^32^. Testosterone also functions as an anti-ecdysteroid, a mechanism of high relevance to arthropods^33^. Eastern mud snail (*Ilyanassa obsoleta*) express NR3C4-like (androgen) and NR3A-like (estrogen) receptors, and exhibit changes in reproductive tract development that are typically associated with androgen or estrogen signaling in vertebrates when exposed to EDCs^33^. The effects of a mixture of flutamide and testosterone on rotifer mixis rate, population growth rate, and resting egg production in the current study demonstrates that these compounds inhibit rotifer reproduction. These results also support the existence of androgen receptors in the endocrine system of rotifers. The ubiquity of certain androgen biotransformation processes in invertebrates reveals differences in the androgen metabolic pathway between this group and vertebrates^30^. Therefore, there might also be an unknown hormone receptor (androgen or otherwise) that binds to metabolites of testosterone and affects endocrine function in the rotifer.

Janer *et al*.^34^ found that vertebrate steroids were also presented in arthropods. And testosterone was presented as an anti-degrading steroid hormone, but the exact function was not clear^32^. Thornton *et al*. isolated the estrogen receptor (ER) of the invertebrates from the mollusks, and found that endocrine signaling pathways are also present in low-grade organisms from the perspective of systemic biology^35^. In addition to estrogen receptor ER, the androgen receptor (AR) also belongs to the nuclear receptor NR3C family from the point of phylogeny. The mechanisms of this receptor are as follows: androgen receptor binding, and the establishment of the target gene between the establishment of a direct signal connection, and ultimately produce the corresponding physiological and biochemical effects. The number of receptors present in other aquatic species (such as *Ciona*) and the NR3C gene repeats have been identified. However, it’s unknown in rotifers, which requires further study.

The presence of steroid hormone has been reported in the female and male individuals of *B. manjavacas*. Since the gene fragment of transcription group in rotifers is not complete. There is no report of the presence of male hormone receptors. At present, the specific mechanism of male hormone in rotifers is not clear. Snell isolated a protein, induced by sexual reproduction, with a molecular weight of 39 kDa from the *B. plicatilus* ^36^. Its N-terminal has 17 amino acid estrus, which is consistent with the steroid-induced protein isolated from human ovarian follicular fluid. And they speculate that the substance may be combined with membrane receptors to produce a series of biological effects, lead to the replacement of asexual reproduction by sexual reproduction. In this study, the concentration of testosterone and flutamide in the present experiment showed that antagonism was observed in the r value, MR, FR, and RE. It is speculated that there may also be a kind of male hormone receptor (AR) and male hormone interaction in the rotifer, which can act on the target gene by signal transduction, and finally interfere with the reproductive system. The existence of the receptor and the specific molecular structure should be studied further.

## Conclusion

This study has demonstrated that testosterone has little effect on asexual reproduction, but has a significant effect on sexual reproduction, in *B. calyciflorus*. Flutamide was more potent than testosterone in the inhibition of reproduction in *B. calyciflorus*. This work also demonstrated that a mixture of flutamide and testosterone had an additive inhibitory effect on reproduction in *B. calyciflorus*. Long-term exposure to either testosterone, flutamide, or a combination of these two compounds may significantly reduce resting egg production in rotifers. This implies that resting egg production is affected differently by hormone pathways and may represent differences in the regulation of rotifer reproduction. The additive toxicity demonstrated in this study suggests a potential ecological risk that requires further study.
